# Supplementary material for: Fast surface reconstruction algorithm with adaptive step size
Source: PLoS One. 2025 Jan 27;20(1):e0314756. doi: 10.1371/journal.pone.0314756 (PMC11771931; doi:10.1371/journal.pone.0314756)
Supplement: Attachment — Submitted filename: Reviewer Reports-kewang.pdf [file pone.0314756.s002.pdf]

Reviewer Reports

In this paper, the authors propose and investigate a surface reconstruction algorithm with an adaptive step size to enhance the computational efficiency of their previous work on Anderson-accelerated implicit progressive iterative approximation (I-PIA). Specifically, the step size is adjusted based on the current residual: larger residuals suggest the need for larger step sizes, while smaller residuals permit smaller step sizes.

The findings presented are both new and intriguing, and I am confident in the rigor of the analysis and numerical experiments. However, a notable weakness of this paper is that it does not offer substantial new insights. If the "adaptive step size" innovation is deemed sufficient for PLOS ONE, I will endorse the acceptance of this paper for publication, contingent upon a thorough review of its formatting, including things like line indents and punctuation.

Kewang Chen,
Ph.D. Mathematical Sciences,
Delft University of Technology, Delft, the Netherlands.
Nanjing University of Information Science and Technology, Nanjing, China
Email: kwchen@nuist.edu.cn
