# Supplementary material for: Fast surface reconstruction algorithm with adaptive step size
Source: PLoS One. 2025 Jan 27;20(1):e0314756. doi: 10.1371/journal.pone.0314756 (PMC11771931; doi:10.1371/journal.pone.0314756)
Supplement: Attachment — Submitted filename: Reply2.pdf [file pone.0314756.s003.pdf]

Dear Editor,

Thank you again for arranging this review process. We also would like to thank the three reviewers for their worthy comments and suggestions on our manuscript. We have revised our manuscript after reading the suggestions provided by the reviewers. We listed below the detailed changes that we have made in the revised manuscript in response to the reviewers' suggestions. We hope that the revised manuscript has addressed all of the reviewers' suggestions. Thank you for your time !

Yours sincerely,
Chengzhi Liu

Below we respond point-by-point to the referees' comments and describe the modifications that we have made in the revised manuscript. We also highlight the changes to our manuscript within the document by using the red, blue, and cyan text, respectively.

Reviewer ♯1: In this study, authors have proposed adaptive method for step size selection in surface reconstruction algorithm. This article is interesting but needs to be revised by considering the following points.

1. There is some inconsistency in section list (e.g As per the last part of introduction section, Numerical examples section must be given number 5. Similarly conclusion part is on number 6). Moreover, needs to be revised for correcting grammatically and typo mistakes.

Our response: We have carefully reviewed the entire document and corrected the section numbering to ensure consistency and logical progression. Besides, we have conducted a thorough proofreading of the manuscript, focusing on grammatical accuracy and typographical precision.

2. Suggest to add preliminaries about adaptive step size in basic section.

Our response: We have added some contents about the adaptive step size in section 3.1.

3. This article is based on adaptive step size, so needs to be add some recent articles relevant to adaptive step size in introduction section.

Our response: Thank you for your insightful feedback. We have added references to several recent articles that are closely related to adaptive step size strategies.

4. It would be better if you define comparison of various algorithms (Iterations & computational complexity) in the form of continuous graph.

Our response: Thank you for your suggestion. We have added a figure (Fig 1) to compare the fitting error vs number of iterations.

5. To facilitate reproducible research, if possible, I suggest that the authors release the related source codes on github.com, the website of the authors' research group, or a similar website. This could make a positive impact on the academic community.

Our response: Thank you for your suggestion. Since organizing the data and code for publication will take some time. We will created a GitHub repository to host the source code associated with our study in the following few week. This repository will include all the scripts, functions, and datasets used in our experiments, ensuring that others can easily access and reproduce our results. More important, our account in GitHub is unable to open recently, see Fig 0.1.

6. Needs to be add future motivation in the last paragraph of conclusion.

Our response: In response to your recommendation, we have expanded the final paragraph of our conclusion to include a discussion on future motivations and directions for our research.

Reviewer ♯2: In this paper, the authors propose and investigate a surface reconstruction algorithm with an adaptive step size to enhance the computational efficiency of their previous work on Anderson-accelerated implicit progressive iterative approximation (I-PIA). Specifically, the step size is adjusted based on the current residual: larger residuals suggest

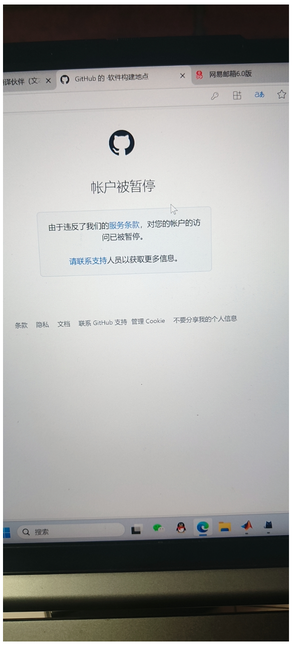

Figure 0.1: Github

the need for larger step sizes, while smaller residuals permit smaller stepsizes.

The findings presented are both new and intriguing, and I am confident in the rigor of the analysis and numerical experiments. However, a notable weakness of this paper is that it does not offer substantial new insights. If the "adaptive step size" innovation is deemed sufficient for PLOS ONE, I will endorse the acceptance of this paper for publication, contingent upon a thorough review of its formatting, including things like line indents and punctuation.

Our response: We appreciate your recognition of the novelty and interest of our findings regarding the adaptive step size surface reconstruction algorithm, which is designed to enhance the computational efficiency of our previous work on the Anderson-accelerated implicit progressive iterative approximation (I-PIA).

We also understand your concern about the paper not offering substantial new insights beyond the adaptive step size innovation. We believe that the dynamic adjustment of the step size, which directly responds to the current residual, is a significant contribution that can lead to more efficient and accurate surface reconstructions, especially in the presence of noise or large datasets. We have refined the conclusion section, where we now clearly delineate the contributions of our paper.

Reviewer ♯3: In this paper a surface reconstruction algorithm is proposed. It is an accelerated version of the I-PIA method of the author as given in Reference 1. The new part is an adaptation of the stepsize of the used Anderson Acceleration method in order to speed up the convergence. From various numerical experiments it follows that the new algorithm is faster than the original method.

Comment

After minor revision this manuscript is suitable for publication in PLOS ONE. Motivation for this recommendation is given below.

Remarks:

1. I miss a couple of references to the literature, please add these in the revised manuscript:

- please give a reference to the paper where the Anderson Acceleration method is proposed first:

D.G. Anderson, Iterative procedures for nonlinear integral equations, J. ACM 12 (1965) 547-560

- please give references to papers where variable Anderson is used:

D.G.M. Anderson, Comments on "Anderson acceleration, mixing and extrapolation", Numer. Algorithms 80 (1) (2019) 135-234

F.A. Potra, H. Engler, A characterization of the behavior of the Anderson acceleration on linear problems, linear Algebra Appl. 438 (3) (2013) 1002-1011

K. Chen and C. Vuik, Non-stationary Anderson acceleration with optimized damping Journal of Computational and Applied Mathematics, 451, 116077, 2024

Our response: We have added several key references.

2. 4 lines below equation (6): is the matrix B^TB non-singular? If yes please give a prove, if no how should singular systems be handled?

Our response: Thank you for your insightful question regarding the singularity of the matrix B^TB. As stated in Property 2 of the convergence analysis section of Reference [13], the matrix B^TB is indeed singular, The I-PIA method (in Reference [13]) effectively handles the singularity of B^TB by solving a constrained minimization problem, ensuring that the solution obtained is the one with the minimum Euclidean norm. This approach not only avoids the issue of infinite solutions but also provides a stable and efficient way to reconstruct the implicit curve and surface. Our method aims to improving the convergence of I-PIA. We hope this explanation clarifies the handling of singular systems in our method. If you have any further questions or require additional details, please do not hesitate to ask.

3. 3 lines below equation (9) the relation with an iterative methods is mentioned. I think that the Richardon iteration method is ment. If yes please give a reference, otherwise indicate which method is ment.

Our response: Thank you for your attention to detail. You are correct in identifying that the relation mentioned three lines below Equation (9) pertains to the Richardson iteration method. We have added the reference.

4. One line below equation (15): what happens if matrix Omega is not of full rank?

Our response: Thank you for your insightful comments. When the matrix Ω is of full rank, the method to handle this situation is by solving a least squares problem to compute the Anderson mixing. Even if the matrix Ω is not of full rank, we can still use Ω to calculate Anderson mixing because in this case, we can find the best Ω vector by using the Moore-Penrose pseudoinverse to a least squares problem, i.e., we can can provide a solution even

if $\Omega^T\Omega$ is singular or nearly singular. Therefore, even if Ω is not of full rank, Anderson mixing can still be effectively calculated and applied.

5. Typo: section Numerical Examples should be Section 5.

Our response: We have revised.

6. Which programming language is used for the experiments?

Our response: We conducted the experiments using MATLAB, and explained in the revised paper.
