# Supplementary material for: Fast surface reconstruction algorithm with adaptive step size
Source: PLoS One. 2025 Jan 27;20(1):e0314756. doi: 10.1371/journal.pone.0314756 (PMC11771931; doi:10.1371/journal.pone.0314756)
Supplement: Attachment — Submitted filename: reviewer-file(Rao8080).docx [file pone.0314756.s001.docx]

**Title:- Fast Surface Reconstruction Algorithm with Adaptive Step Size**

**Reviewer:- In this study, authors have proposed adaptive method for step size selection in surface reconstruction algorithm. This article is interesting but needs to be revised by considering the following points.**

**1. There is some inconsistency in section list (e.g As per the last part of introduction section, Numerical examples section must be given number 5.Similarly conclusion part is on number 6). Moreover, needs to be revised for correcting grammatically and typo mistakes.**

**2. Suggest to add preliminaries about adaptive step size in basic section.**

**3. This article is based on adaptive step size, so needs to be add some recent articles relevant to adaptive step size in introduction section.**

**4. It would be better if you define comparison of various algorithms (Iterations & computational complexity) in the form of continuous graph.**

**5. - To facilitate reproducible research, if possible, I suggest that the authors release the related source codes on github.com, the website of the authors' research group, or a similar website. This could make a positive impact on the academic community.**

**6. Needs to be add future motivation in the last paragraph of conclusion.**
